# Self-Polymerized Dopamine Nanoparticles Modified Separators for Improving Electrochemical Performance and Enhancing Mechanical Strength of Lithium-Ion Batteries

**DOI:** 10.3390/polym12030648

**Published:** 2020-03-12

**Authors:** Wenqian Hao, Dechong Kong, Jiamiao Xie, Yaping Chen, Jian Ding, Fenghui Wang, Tingting Xu

**Affiliations:** 1 Bi-inspired and Advanced Energy Research Center, Department of Engineering Mechanics, Northwestern Polytechnical University, Xi’an 710129, China; wqhao@mail.nwpu.edu.cn (W.H.); ypchennwpu@163.com (Y.C.); dingjian@mail.nwpu.edu.cn (J.D.); 2 Department of Applied Chemistry, School of Science, Northwestern Polytechnical University, Xi’an 710129, China; kdc1017@163.com; 3 College of Mechanical and Electrical Engineering, North University of China, Taiyuan 030051, China; xiejiamiao618@163.com

**Keywords:** self-polymerized dopamine, macromolecular separators, electrochemical performance, mechanical strength, lithium-ion batteries

## Abstract

Separators in lithium-ion batteries (LIBs) play an important role for battery safety, so stable electrochemical performance and high mechanical strength of separators will always be of interest. On the basis of the fact that polydopamine (PDA) nanoparticles found in mussel have a strong adhesion ability, biomaterial surface nanoparticles modification methods are developed to increase electrochemical performance and enhance mechanical strength of polypropylene (PP) and polypropylene/polyethylene/polypropylene (PP/PE/PP) separators. The electrolyte uptake performance, ionic conductivities, discharging rate capabilities, yield stresses, and failure strains of PP and PP/PE/PP separators are all enhanced remarkably by PDA modification. Thermal shrinkage results show that thermal stabilities and the shrinkage percentage of PDA-modified separators are improved. The electrochemical testing results conclude that the discharging capacities of PP (increased by 3.77%~187.57%) and PP/PE/PP (increased by 2.31%~92.21%) separators increase remarkably from 0.1 C to 5.0 C. The ionic conductivities of PDA-modified PP and PP/PE/PP separators are 1.5 times and 6.1 times higher than that of unmodified PP and PP/PE/PP separators, which in turn increase the electrolyte uptake and ionic migration. In addition, mechanical properties of PP (yield stresses: 17.48%~100.11%; failure stresses: 13.45%~82.71%; failure strains: 4.08%~303.13%) and PP/PE/PP (yield stresses: 11.77%~296.00%; failure stresses: 12.50%~248.30%; failure strains: 16.53%~32.56%) separators are increased greatly.

## 1. Introduction

Lithium-ion batteries (LIBs) have been widely used in electrochemical energy conversion and storage for a wide range of applications, including electronic devices, electric vehicles and their energy systems, and so forth, due to their high energy density, long cycle life, zero memory effect, low self-discharge, and environmental friendliness [1,2,3,4,5,6,7]. LIBs mainly consist of positive electrodes, negative electrodes, electrolytes, and macromolecular separators. The separators are placed between positive and negative electrodes to protect against an internal short circuit and provide favorable channels for lithium ions migration [8]. When separators suffer mechanical failure under external loading, the lithium dendrites may puncture the separators, leading to an internal short circuit or even the explosion of the batteries, due to direct contact between positive and negative electrodes [9,10]. Although micro-porous single-layer polypropylene (PP) or tri-layer polypropylene/polyethylene/polypropylene (PP/PE/PP) separators have been adopted in commercial LIBs, research concerning the stable electrochemical performance and high mechanical strength of separators remains an important research issue.

However, conventional polyolefin separators are intrinsically poorly compatible with electrolytes, due to their hydrophobic surface character and low surface energy, which result in low electrolyte uptake and poor lithium ion conductivity [11,12,13,14]. In addition, many external conditions also affect the performance of materials, such as freezing, heat, physical design, and chemical corrosion [7,11,15,16,17]. To overcome the poor hydrophilicity of polyolefin separators, many efforts have been made to characterize the electrochemical performances and mechanical properties, such as the plasma modified graft [18], ultraviolet irradiation [19], and electron beam irradiation [20]. However, these modified methods require expensive equipment and complex procedures. In addition, the previously employed strategy (such as nano-silica particles, sodium dihydrogen citrate, and isopropyl alcohol) not only lowers the mechanical strength of separators but also leads to safety issues of LIBs because of the non-uniform pore sizes in the separators. On the basis of the fact that polydopamine (PDA) found in mussel has a strong adhesion ability, biomaterial surface nanoparticles modification methods were developed to increase the surface hydrophilicity and enhance the mechanical strength of different materials [21,22]. The PDA solution was obtained by self-polymerized behavior of dopamine in alkaline buffer solution, which was easy to operate and free from expensive equipment, compared to other methods [23,24,25]. In addition, there is little research about PDA surface modification of PP and PP/PE/PP separators for LIB in recent years.

Therefore, the purpose of the present research is to deposit PDA nanoparticles on the surface and micro-porous edge of PP and PP/PE/PP separators, so as to improve the electrochemical performance and mechanical strength of separators. The research offers several highlights, such as that (1) the thermal shrinkage and electrolyte uptake performance of PP and PP/PE/PP separators are improved by PDA surface nanoparticles modification method; (2) the electrochemical performances (ohmic resistance decreases and ionic conductivity increases) of separators are improved remarkably; (3) the mechanical properties (yield stresses, failure stresses and failure strains) of PP and PP/PE/PP separators along different directions are enhanced greatly by PDA modification method. The research provides guidance for improving the manufacturing procedure, exploring the mechanical property, and addressing the safety issue of separators in LIBs.

## 2. Experimental

### 2.1. Polydopamine Modified Separator Preparation

In order to investigate the effect of polydopamine (PDA) surface modification on the LIB separator, two typical macromolecular separators were selected, one was a single-layer polypropylene (PP) separator (thickness: 25 μm; porosity: 55%) and the other was a tri-layer polypropylene/polyethylene/polypropylene (PP/PE/PP) separator (thickness: 25 μm; porosity: 39%). The fundamental properties of the separators are summarized in Table 1 [26,27,28]. As a functional material, there was an obvious anisotropic effect of the separator, due to its manufacturing procedure and property difference among machine direction (MD), diagonal direction (DD), and transverse direction (TD).

**Figure 1:**
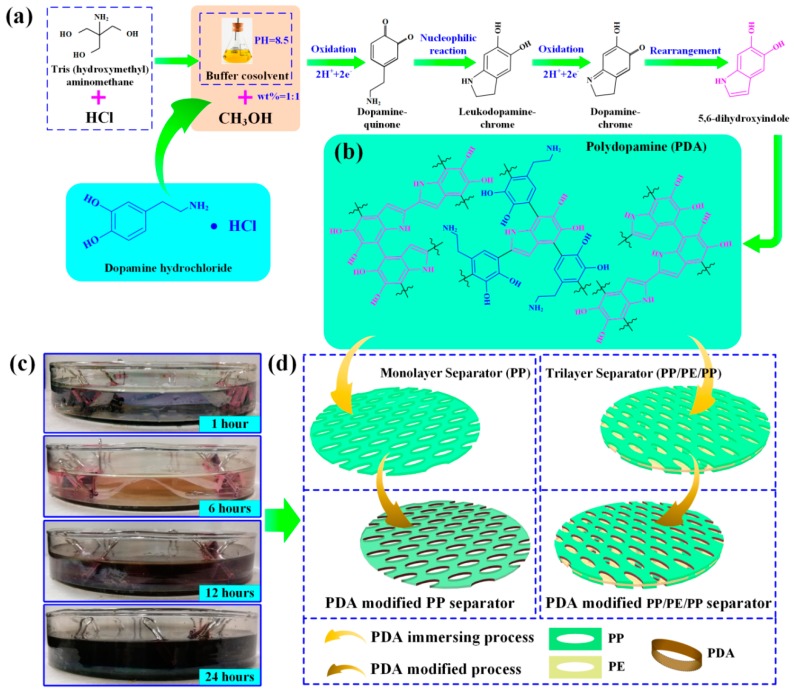
Schematic and preparation of the polydopamine(PDA)-modified separator: (**a**) dopamine polymerization mechanism for PDA; (**b**) chemical structure of PDA; (**c**) images of the PDA-modified separator and dopamine solution change over dipping time; (**d**) schematic illustration of the PDA-modified separator process by immersing method.

The PDA modification of the separator was obtained by immersing the separator into dopamine solution (10 mmol L^−1^) for 24 h at room temperature, as shown in Figure 1. The dopamine solution was prepared by dissolving dopamine hydrochloride (AR, Aladdin Industrial Corporation, Shanghai, China, 98%) in methanol (AR, Aladdin Industrial Corporation, Shanghai, China, 99.5%) and Tris buffer solution with a volume ratio of 1:1. The Tris buffer solution (PH=8.5) was prepared by the ultrasonic mixing of Tris (hydroxymethyl) aminomethane hydrochloride (Tris HCl) (AR, Aladdin Industrial Corporation, Shanghai, China, > 99%) and hydrochloric acid (Figure 1a,b). Firstly, catechol in dopamine was self-oxidized to dopamine-quinone by alkaline PH-induced oxidation [23,24]. Secondly, the nucleophilic reaction of dopamine-quinone occurred to produce leukodopamine-chrome and the solution was colorless and transparent after 1 h, as shown in Figure 1c. Thirdly, the leukodopamine-chrome was self-oxidized to a dopamine-chrome intermediate solution and the solution changed from colorless to pink after 6 h as shown in Figure 1c. Subsequently, the obtained dopamine-chrome solution was rearranged to form 5,6-dihydroxyindole (DHI). Finally, PDA was prepared with a series of dopamine molecules and DHI molecules by non-covalent self-assembly and covalent oxidation polymerization [29]. The structure of PDA was revealed to DHI chemistry, which was similar to melanin biosynthesis [22,30]. Consequently, the solution changed from pink to brown-black after 12 h and finally to black after 24 h as shown in Figure 1c. The PDA-modified separator turned from white to dark-brown during the self-polymerization process of dopamine [22,31].

### 2.2. Materials Characterizations

The microstructure of the sample was observed using field emission scanning electron microscope (FE-SEM, FEI Verios G4, Hillsboro, OR, US) in a vacuum environment with 10 kV and 2 kV accelerating voltage. The thermal shut-down property of the separator was characterized by differential scanning calorimeter (DSC, NETZSCH STA 449 F3 Jupiter, Selb, Germany). About 1.5 mg weight of the sample was tested at the heating or cooling rate of 10 °C min^−1^ under argon atmosphere, and the temperature range was from 25 °C to 700 °C. Thermogravimetric (TG, NETZSCH STA 449 F3 Jupiter, Selb, Germany) analysis was conducted in argon atmosphere from 25 °C to 700 °C, at a heating or cooling rate of 10 °C/min. The nature of the sample of the separator was identified by X-ray diffractometer (Shimadzu XRD-7000, Kyoto, Japan) with monochromatic 3 kW Al K*α* radiation, and the scan range was from 5° to 85°. The elemental composition was measured by X-ray photoelectron spectroscopy (XPS, Kratos Axis Supra, Manchester, UK) with monochromatic 150 W Al K*α* radiation. The electrolyte uptake percentage *α* was calculated according to the equation [4,7]

(1) $$\alpha =\frac{W_{a}-W_{b}}{W_{b}}\times 100\%$$

where *α* is the electrolyte uptake percentage, *W_b_* is the weight of dry sample, and *W_a_* is the weight after being soaked in the electrolyte. The weight of dry and soaked samples was measured by precision electronic balance.

### 2.3. Electrochemical Performance Experiments

**Figure 2:**
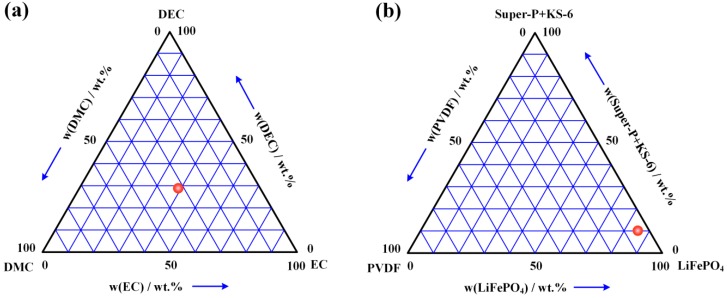
The volume ratio of electrolyte and positive electrode: (**a**) mixture of ethylene carbonate (EC), dimethyl carbonate (DMC) and diethyl carbonate (DEC); (**b**) mixture of LiFePO_4_, conductive carbons (Super-P Li and KS-6) and binder poly(vinylidene difluoride) (PVDF).

The electrochemical performance of the PDA-modified separator was tested through assembling CR2025 coin type cell in an argon-filled glove box. The CR2025 coin type cell contained positive electrodes (LiFePO_4_), an electrolyte-soaked with or without the PDA-modified separator, and a lithium foil negative electrode. The electrolyte used in the experiment was papered by dissolving 1 mol L^−1^ LiPF_6_ (Tech, Aladdin Industrial Corporation, Shanghai, China, >97 %) in a mixture of ethylene carbonate (EC) (AR, Aladdin Industrial Corporation, Shanghai, China, 98 %), dimethyl carbonate (DMC) (AR, Aladdin Industrial Corporation, Shanghai, China, >98 %) and diethyl carbonate (DEC) (AR, Aladdin Industrial Corporation, Shanghai, China, 99 %) with a volume ratio of 1:1:1, as shown in Figure 2a. The positive electrode was prepared by mixing 85 wt% active materials LiFePO_4_ (Aleees, Advanced Lithium Electrochemistry Co., Ltd., Taiwan, China), 10 % conductive carbons Super-P Li and KS-6 (TIMCAL, TIMCAL Ltd., Bodio, Switzerland), 5 % binder poly(vinylidene difluoride) (PVDF) (Kynar 741, Arkema Inc., Colombes, France), as shown in Figure 2b. The solvent used in the positive electrode was N-methylpyrrolidone (NMP) (AR, Aladdin Industrial Corporation, Shanghai, China, 98 %). After 24 h of stirring, homogeneous positive electrode slurry was formed and cast onto the aluminum foil using the scraper knife. Then, the positive electrode and aluminum foil were dried at 100 °C for 12 h in vacuum environment. The charge/discharge performance and ionic conductivity were analyzed with battery test equipment (LAND CT2001A, Wuhan LAND Electronic Co, Ltd., Wuhan, China) and an electrochemical workstation (CHI 660E, Shanghai Chenhua Instrument Co, Ltd., Shanghai, China). The unit cell was pre-cycled from 3.0 V to 4.5 V at the voltage scanned rate of 1 mV s^−1^.

Ionic conductivity was obtained by electrochemical impedance spectroscopy (EIS) of the electrolyte-soaked separator between two stainless steel (SS) electrodes (contact area S=3.14 cm^2^) at an open-circuit potential in a frequency from 1 MHz- 1 Hz. The ionic conductivity κ is given

(2) $$\kappa =\frac{t}{R_{o}S}$$

where *t* is the thickness of separators, *R*_o_ is Ohmic resistance, and *S* is the contact area between separators and stainless steel (SS) electrodes.

### 2.4. Mechanical Strength Experiments

To ensure the consistency of the sample geometry, a polymethyl methacrylate (PMMA) template with 0.5 mm thickness was milled. The sample was cut along the perimeter of the template on transparent Cartesian graph paper to improve the dimensional accuracy and cut quality. According to the ASTM D638 tension testing standard [26] for polymer material, a mechanical strength experiment was performed using an electronic universal testing machine (SANS CMT 4104, Shenzhen SANS Test Machine Co., Ltd., Shenzhen, China) with a 500 N load cell (accuracy: 0.005 N) [6]. The two ends of the sample were glued with rubber to prevent sliding and tearing and then they were held by the two flat grips of the testing machine. Uniaxial tension experiments under three tension strain rates (0.002 s^−1^, 0.02 s^−1^ and 0.2 s^−1^) were carried out at room temperature. The length and width of the specimen was 80 mm and 20 mm, respectively. The gauge length was chosen as 35 mm and at least three identical samples were tested. The strain rate is the change rate of strain with time and it is given by

(3) $$\dot{\varepsilon }=\frac{d\varepsilon }{dt}=\frac{v_{0}}{l_{0}}$$

where *v*_0_ is the initial loading velocity of testing machine and *l*_0_ is the initial gauge length of separator.

## 3. Results and Discussion

**Figure 3:**
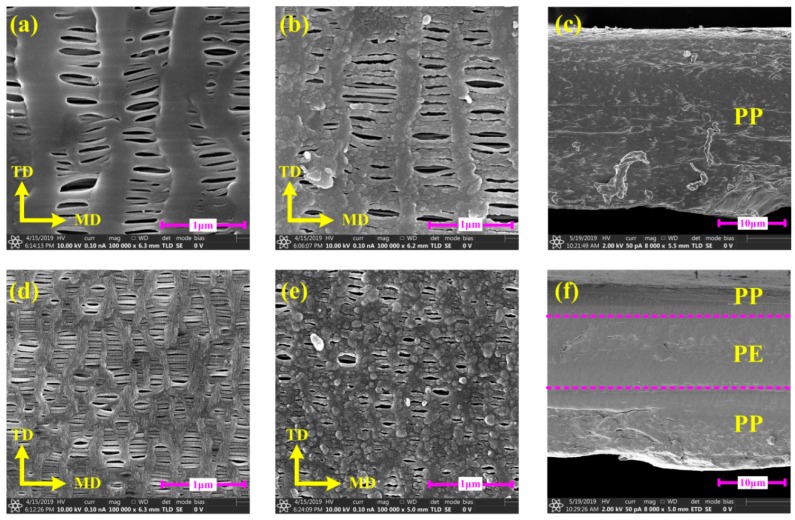
Field emission scanning electron microscope (FE-SEM) images of unmodified and PDA-modified separators on the surface and cross-section: (**a,b**) unmodified and PDA-modified polypropylene (PP) separators on the surface; (**c**) unmodified PP separator on the cross-section; (**d,e**) unmodified and PDA-modified polypropylene/polyethylene/polypropylene (PP/PE/PP) separators on the cross-section; (**f**) unmodified PP/PE/PP separator on the cross-section.

In order to investigate the effect of PDA nanoparticles modification on the morphologies of separators, the surface (Figure 3a,b,d,e) and cross-section (Figure 3c,f) microstructures of unmodified and PDA-modified separators were analyzed by extra-high resolution field emission scanning electron microscope (FE-SEM). From the microcosmic point of view, the anisotropic effect occurred obviously because of the rearrangement of macromolecular chains and chain segments caused by lattice orientation (Figure 3a,d). In addition, the lamellae and fibrils of each separator in the surface morphology became stronger after PDA modification, although some of the pores were blocked (Figure 3b,e).

**Figure 4:**
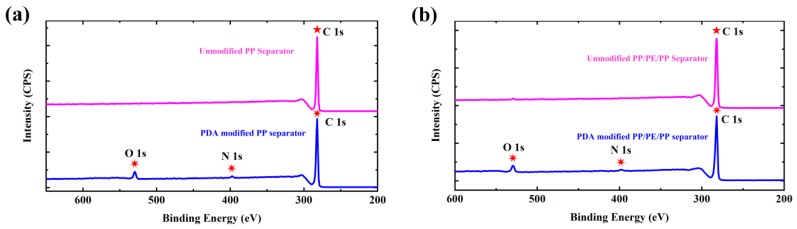
High-resolution X-ray photoelectron spectroscopy (XPS) spectra of separators (including C 1s, O 1s and N 1s): (**a**) unmodified and PDA-modified PP separators; (**b**) unmodified and PDA-modified PP/PE/PP separators.

The fundamental characterizations of the unmodified and PDA-modified separators were displayed. As illustrated in Figure 4, the surface composition changes of separators resulted from PDA modification were confirmed by X-ray photoelectron spectroscopy (XPS) spectra. The unmodified PP and PP/PE/PP separators exhibited only C 1s peaks, while PDA-modified PP and PDA-modified PP/PE/PP separators newly appeared N 1s and O 1s peaks. Therefore, it was indicated that the separators’ surfaces were coated by PDA-modified particles (Figure 3b,e).

**Figure 5:**
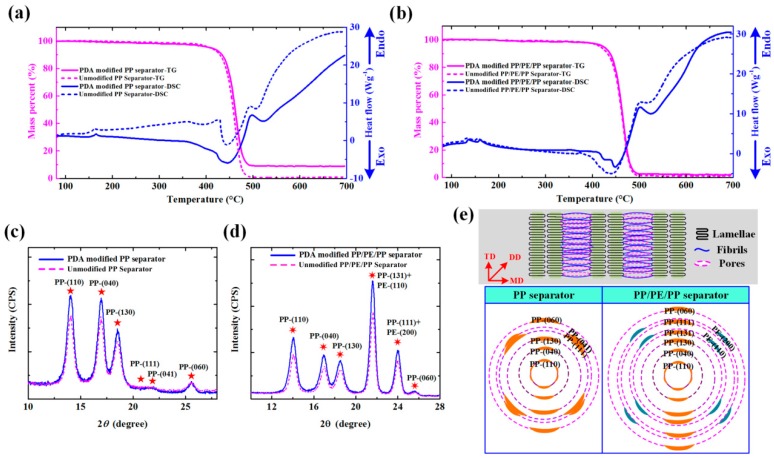
Characterizations of unmodified and PDA-modified separators: (**a,b**) Thermogravimetric (TG) and differential scanning calorimetric (DSC) curves of unmodified PP, PDA-modified PP, unmodified PP/PE/PP, and PDA-modified PP/PE/PP separators; (**c,d**) X-ray diffractometer (XRD) spectra of unmodified PP, PDA-modified PP, unmodified PP/PE/PP, and PDA-modified PP/PE/PP separators; (**e**) diffraction schematic diagram of lattice plane distributions of the PP separator indicated by PP-(110), PP-(040), PP-(130), PP-(111), PP-(041), and PP-(060) and the PP/PE/PP separator indicated by PP-(110), PP-(040), PP-(130), PP-(131)+PE-(110), PP-(111)+PE-(200), and PP-(060).

The thermal properties of the unmodified and PDA-modified separators were measured by thermogravimetric (TG) and differential scanning calorimetric (DSC) analyses, respectively (Figure 5a,b). The unmodified PP, PDA-modified PP, unmodified PP/PE/PP and PDA-modified PP/PE/PP separators started to break down at 418.5 °C, 426.7 °C, 421.2 °C and 430.6 °C and remaining quantities were 1.16%, 10.80%, 2.28% and 3.58%, respectively. It meant that PDA-modified method could improve the thermal stabilities of separators. Shrinkage percentage is the capability to shut down the microporous of separator before thermal runaway occurs and it plays an important role in application of LIBs. When shrinkage temperature is close to melting temperature *T_m_*, the microporous layer turns into non-porous insulating layer that (i) prevents the direct contact between positive and negative electrodes and (ii) reduces the conductivity and electrochemical activity between separator and electrolyte [32]. Through PDA-modified method, the melting temperature *T_m_* of PP separator increased from 167.1 °C to 169.0 °C, while PP/PE/PP separator increased from 136.9 °C to 140.6 °C for PE layer and from 160.4 °C to 165.2 °C for PP layer. With separators began to break down between 400 °C and 500 °C (see TG curves), the exothermic reaction including solidification, oxidization, reaction and crosslinking were taken place (see DSC curves). The crystallinities of separators were characterized by X-ray diffractometer (XRD), as shown in Figure 5c,d. The PP separator was monoclinic [33] and belonged to P2_1_/c(No. 14) space group, diffraction peaks of PP-(110) and PP-(040), PP-(130), PP-(111), PP-(041) and PP-(060) occurred at 2*θ* = 14.1°, 17.0°, 18.6°, 21.2°, 22.0° and 25.6° [34,35,36]. The PP/PE/PP separator was the compound of PP and PE, so the diffraction peaks of PP-(131)+PE-(110) and PP-(111)+PE-(200) overlapped at 2*θ* = 21.6° and 24.0° in Figure 5d because PE separator was orthorhombic and belonged to *P_nam_*(No. 62) space group. Because of the size effects and surface effects of the PDA nano-particle coating, the diffraction and emission peaks were broadened. This indicated that the dopamine-modified particles could reduce the crystallinity and increase the proportion of amorphous crystalline. So more electrolyte could be absorbed and ionic conductivity of separator was improved. The diffraction schematic diagrams of lattice plane distributions were obtained, due to anisotropic effect of separator (Figure 5e). As the most stable crystal form of *α*-PP crystals exhibited a unique lamellar branching structure, the lattice plane distributions of lamellae (mother crystal) and fibrils (daughter crystal) were different [37,38,39,40]. The diffraction intensity of the separator increased after PDA surface modification. It meant that the crystallinity of the PDA-modified separator was smaller than that of unmodified separator (Figure 5c,d). Efficient electrolyte absorption of the separator is essential for achieving high Li^+^ conductivity and low Ohmic resistance [26,41].

**Figure 6:**
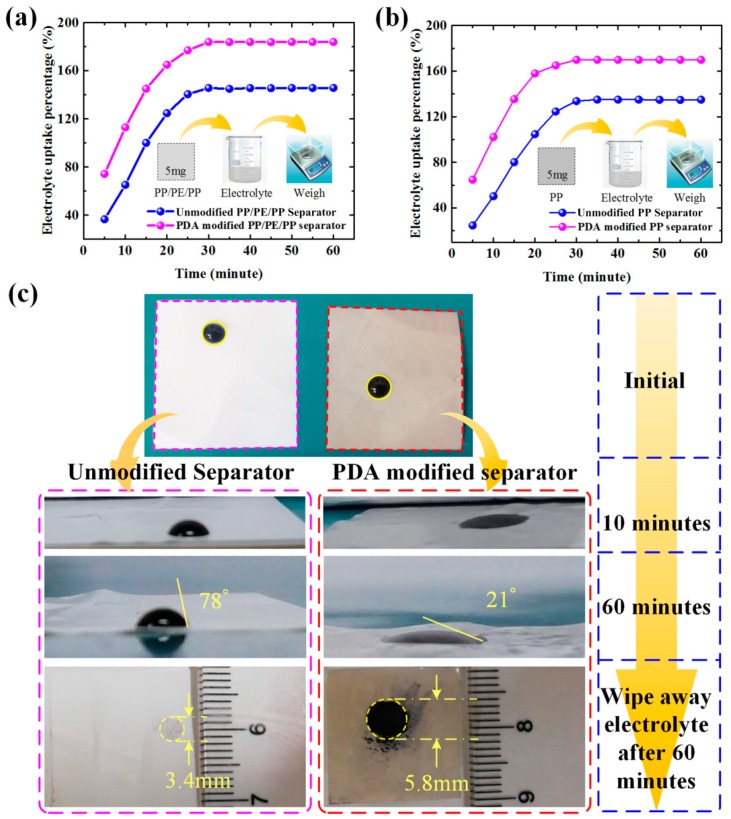
The changes of the electrolyte uptake percentages of separators with time: (**a,b**) unmodified and PDA-modified separators; (**c**) contact between unmodified/PDA-modified separator and colored electrolyte with time, and contact angles of unmodified and PDA-modified separators are 78° and 21°; the colored electrolyte was wiped away after 60 min and contact areas of unmodified and PDA-modified separators were 9.07 mm^2^ and 26.41 mm^2^.

The unmodified and PDA-modified separators (initial weight is *W_b_* = 5 mg) were immersed in the electrolyte for 60 min, as illustrated in Figure 6a,b. The electrolyte uptake percentages *α* were calculated by Equation (1) in Experimental section. Owing to the affinity to the electrolyte and capillary effect of PDA, the electrolyte uptake percentage of the separators modified by PDA (modified PP: *α*_max_=170%; modified PP/PE/PP: *α*_max_=184%) was much higher compared with unmodified PP and PP/PE/PP separators (unmodified PP: *α*_max_=135%; unmodified PP/PE/PP: *α*_max_=145%). Pictures of contact between unmodified/PDA-modified separator and colored electrolyte with time are shown in Figure 6c. The hydrophilic surface and good compatibility of the PDA-modified separator increased the electrolyte uptake, leading to a 21° static contact angle after 60 min, which is far less than that of the unmodified separator (78° contact angle). It was confirmed that the PDA nanoparticles coating obviously improved the hydrophilicity of separator surface. The colored electrolyte was wiped away after 60 min and contact areas of unmodified and PDA-modified separators were 9.07 mm^2^ and 26.41 mm^2^, respectively. Besides, more electrolytes adhered to the surface of PDA-modified separator. Therefore, one potential advantage of the PDA nanoparticles coating was the adhesion improvement.

**Figure 7:**
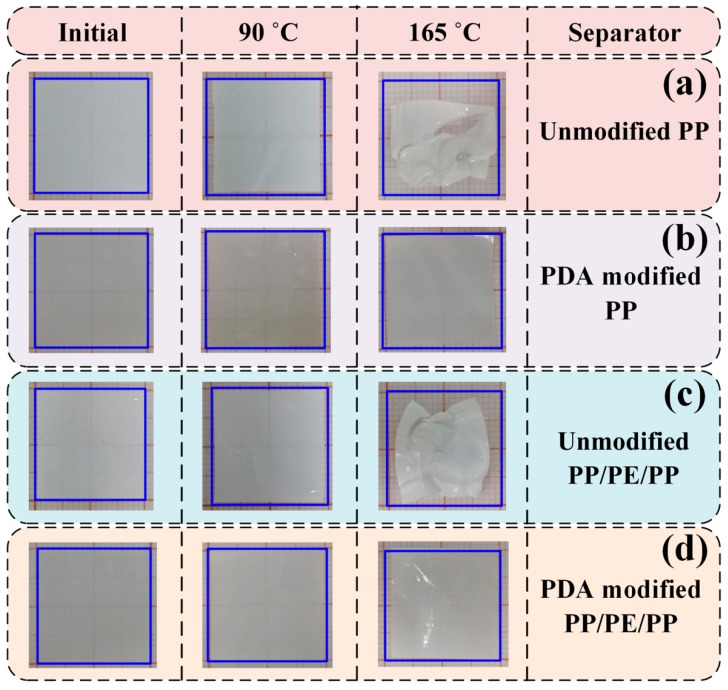
The changes of thermal shrinkages of separators with temperature: (**a**) unmodified PP; (**b**) PDA-modified PP; (**c**) unmodified PP/PE/PP; (**d**) PDA-modified PP/PE/PP.

The long-term stability of separators should be shown by holding them at elevated temperatures and observing the dimensional shrinkage. The changes of thermal shrinkages of unmodified and PDA-modified separators with temperatures were measured, as shown in Figure 7. Two temperatures (90 °C and 165 °C), which are commonly used in the battery assembly process, were selected as the investigated temperatures. After 90 °C treatment, the thermal shrinkages of the four separators did not change much. Furthermore, the thermal shrinkages of PDA-modified separators (modified PP: ≈4.5%; modified PP/PE/PP: ≈2.5%) were slightly smaller than that of unmodified separators (unmodified PP: ≈5.0%; unmodified PP/PE/PP: ≈4.5%). While after 165 °C treatment, the thermal shrinkages of PDA-modified separators (modified PP: ≈10%; modified PP/PE/PP: ≈7.5%) were obviously smaller than that of unmodified separators (unmodified PP: ≈45%; unmodified PP/PE/PP: ≈30%). It meant that the thermal shrinkage of separators could be reduced by PDA-modified method.

**Figure 8:**
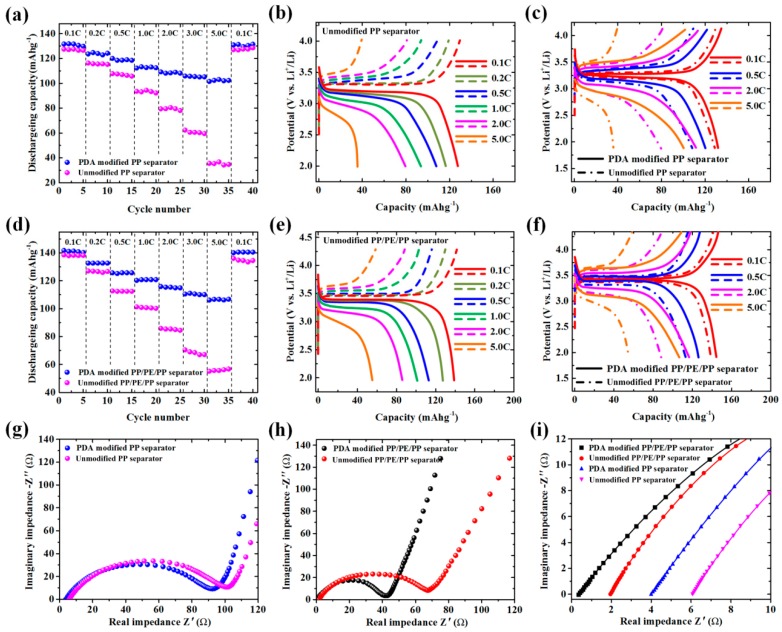
Electrochemical performances of cells with unmodified and PDA-modified separators: (**a,d**) discharging rate capacities; (**b,e,c,f**) galvanostatic charge-discharge curves at different current rates; (**g–i**) electrochemical impedance spectroscopies.

The effect of PDA modification to electrochemical performances was investigated by assembling the CR2025 coin type cell (LiFePO_4_/separator/Li foil). Discharging rate capabilities of unmodified PP, PDA-modified PP, unmodified PP/PE/PP, and PDA-modified PP/PE/PP separators are shown in Figure 8a,d. For the PP separator, the discharging capacities of the unmodified separator (127.3 mAhg^−1^) and PDA-modified separator (132.1 mAhg^−1^) were nearly the same at 0.1 C, but the discharging capacities of the PDA-modified separator (124.6, 119.8, 112.5, 109.0, 105.8, and 101.8 mAhg^−1^) were greater than that of the unmodified separator (116.6, 107.5, 93.5, 79.5, 61.9, and 35.4 mAhg^−1^) from 0.2 C to 5.0 C (Figure 8a). For PP/PE/PP separator, the discharging capacities of unmodified separator (138.7, 126.7, 112.6, 101.3, 85.9, 70.2 and 55.2 mAhg^−1^) and PDA-modified separator (141.9, 132.8, 125.8, 120.4, 115.8, 110.5 and 106.1 mAhg^−1^) were tested from 0.2 C to 5.0 C (Figure 8d). The charge-discharge curves of the unmodified and PDA-modified separators at different current rates are presented in Figure 8b,c,e,f. The PDA-modified separators displayed better voltage platform and greater charge-discharge capacity compared with unmodified separators at different C. The excellent electrochemical performance of the PDA-modified separators was attributed to the improvement of the electrolyte uptake performance and ionic conductivity. Electrochemical impedance spectroscopy (EIS) was analyzed to further investigate the reason for increasing the charge-discharge capacity of cells with PDA-modified separators (Figure 8g–i). The SS/PDA-modified separator/SS had a small semicircle diameter at high frequency compared with SS/unmodified separator/SS, as shown in the Nyquist curves (Figure 8g,h). This indicates that the electrode/PDA-modified separator had lower Li^+^ migration resistance and higher electrochemical reaction kinetics [13]. The straight lines were displayed in the medium and low frequency region among all separators, which was relevant to Li^+^ diffusion resistance between electrode and electrolyte. The ohmic resistances of the PDA-modified separator (PP: 3.844 Ω, PP/PE/PP: 0.296 Ω) were smaller than that of the unmodified separator (PP: 5.876 Ω, PP/PE/PP: 1.825 Ω), as shown in Figure 8i. The ionic conductivity (Equation (2) in Experimental section) of the PDA-modified PP separator could reach *κ* = 0.21 mS cm^−1^, which was 1.5 times higher than that of unmodified PP separator (*κ* = 0.14 mS cm^−1^). The ionic conductivity of PDA-modified PP/PE/PP separator (*κ* = 2.69 mS cm^−1^) was 6.1 times higher than that of unmodified PP/PE/PP separator (*κ* = 0.44 mS cm^−1^). Therefore, the decrease of the ohmic resistance and the increase of ionic conductivity of PDA-modified separator were due to the improvement of the separator surface by the PDA hydrophilic group, which increased the electrolyte uptake and ionic flux.

**Figure 9:**
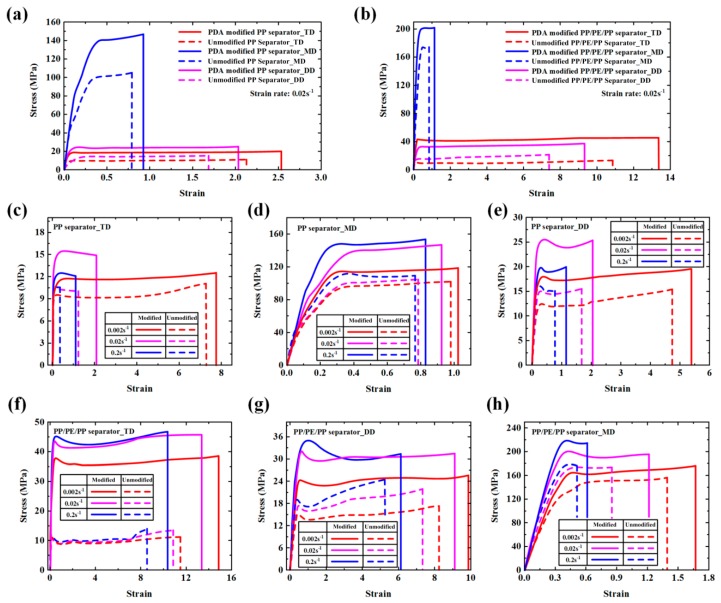
Mechanical properties of unmodified and PDA-modified separators: (**a,b**) stress-strain curves along transverse direction (TD), machine direction (MD), and diagonal direction (DD); (**c–h**) stress-strain curves under three strain rates (0.002 s^−1^, 0.02 s^−1^ and 0.2 s^−1^).

The mechanical properties of the unmodified and PDA-modified separators under uniaxial tension along TD, MD, and DD were also measured, due to the obvious anisotropic effects shown in Figure 9a,b. The yield stresses, failure stresses, and failure strains of unmodified and PDA-modified separators along TD, MD, and DD under three strain rates (0.002 s^−1^, 0.02 s^−1^ and 0.2 s^−1^) are shown in Table 2, Table 3 and Table 4. The yield stresses, failure stresses, and failure strains of PP and PP/PE/PP separators were improved greatly through PDA modification. For the PP separator along TD, MD, and DD under different strain rates, the yield stresses, failure stresses, and failure strains increased by 17.48%~100.11%, 13.45%~82.71 %, and 4.08%~303.13%, respectively. While for the PP/PE/PP separator, the yield stresses, failure stresses, and failure strains increased by 11.77%~296.00%, 12.50%~248.30%, and 16.53%~32.56%, respectively. The reason for this was that the PDA-modified method made lamellae and fibrils stronger and this method did not block the pores (Figure 3b,e). In addition, because the separators were very thin, this modified method does not affect the electrochemical performance. As shown in Figure 9c–h, the obvious strain rate effect of unmodified and PDA-modified separators was observed, and yield stresses, failure stresses, and failure strains increased with the strain rate. The PDA-modified separators along MD had higher strength but lower toughness compared with that along DD and TD (Figure 9i–k).

## 4. Conclusions

The self-polymerized dopamine modified PP and PP/PE/PP separators were prepared for improving the electrochemical performance and enhancing the mechanical strength of LIBs. The TG and DSC results of the separators showed that the thermal stabilities and shrinkage percentages were improved by PDA modification. The PDA-modified particles could also reduce the crystallinity and increase the electrolyte uptake performance of the separators. The electrolyte uptake performance, ionic conductivities, discharging rate capabilities, yield stresses, and failure strains of the PP and PP/PE/PP separators were all enhanced remarkably by PDA modification. Thermal shrinkage results show that thermal stabilities and the shrinkage percentage of PDA-modified separators were improved. The electrochemical testing results conclude that the discharging capacities of PP (increased by 3.77%~187.57%) and PP/PE/PP (increased by 2.31%~92.21%) separators increased remarkably from 0.1 C to 5.0 C, once the application of these separators was in the battery systems. The ionic conductivities of PDA-modified PP and PP/PE/PP separators were 1.5 times and 6.1 times higher than that of unmodified PP and PP/PE/PP separators, which in turn increased the electrolyte uptake and ionic migration. In addition, the mechanical properties of PP (yield stresses: 17.48%~100.11%, failure stresses: 13.45%~82.71%, failure strains: 4.08%~303.13%) and PP/PE/PP (yield stresses: 11.77%~296.00%, failure stresses: 12.50%~248.30%, failure strains: 16.53%~32.56%) separators were enhanced greatly through PDA modification.

## Figures and Tables

**Table 1:** Fundamental properties of single-layer polypropylene (PP) and tri-layer polypropylene/ polyethylene/ polypropylene (PP/PE/PP) separators [26,27,28].

Separators	Thickness (µm)	Permeability (s)	Porosity (%)	PP Pore Size (µm)	Tensile Strength (MPa)		Puncture Strength (N)	Thermal Stability (Shrinkage) 90°C/1h (%)	
					TD	MD		TD	MD
Single-layer (PP)	25	200	55	0.064	13.24	103.46	>3.283	0	<5
Tri-layer (PP/PE/PP)	25	620	39	0.028	14.71	166.71	>3.724	0	<5

**Table 2:** Material parameters of unmodified and PDA-modified separators along transverse direction (TD), machine direction (MD), and diagonal direction (DD) under strain rates 0.002 s^−1^.

Material Parameters	Yield Stress (MPa)			Failure Stress (MPa)			Failure Strain		
Tension Direction	MD	TD	DD	MD	TD	DD	MD	TD	DD
Unmodified PP	95.92	9.55	12.42	102.21	11.08	15.42	0.98	7.24	4.78
PDA-modified PP	114.40	11.69	17.97	118.33	12.57	19.54	1.02	7.72	5.41
Unmodified PP/PE/PP	147.69	11.22	15.17	156.38	11.22	17.52	1.40	11.47	8.24
PDA-modified PP/PE/PP	165.07	37.91	24.41	175.93	38.67	25.63	1.67	14.87	9.86

**Table 3:** Material parameters of unmodified and PDA-modified separators along TD, MD, and DD under strain rates 0.02 s^−1^.

Material Parameters	Yield Stress (MPa)			Failure Stress (MPa)			Failure Strain		
Tension Direction	MD	TD	DD	MD	TD	DD	MD	TD	DD
Unmodified PP	100.22	9.42	14.30	105.45	10.93	15.52	0.79	2.13	1.69
PDA-modified PP	140.74	18.85	24.79	147.03	19.97	25.61	0.93	2.54	2.03
Unmodified PP/PE/PP	174.83	10.98	15.10	174.83	13.27	21.97	0.86	10.86	7.39
PDA-modified PP/PE/PP	201.37	43.48	32.95	202.75	46.22	37.53	1.14	13.37	9.32

**Table 4:** Material parameters of unmodified and PDA-modified separators along TD, MD, and DD under strain rates 0.2 s^−1^.

Material Parameters	Yield Stress (MPa)			Failure Stress (MPa)			Failure Strain		
Tension Direction	MD	TD	DD	MD	TD	DD	MD	TD	DD
Unmodified PP	112.04	10.64	16.14	109.68	10.59	15.03	0.77	0.32	0.95
PDA-modified PP	148.21	12.50	19.87	153.71	12.06	20.00	0.83	1.29	1.30
Unmodified PP/PE/PP	178.12	12.31	19.09	178.84	13.83	24.76	0.52	8.58	5.26
PDA-modified PP/PE/PP	218.82	45.21	35.13	213.94	46.84	31.47	0.62	10.36	6.13
